# Robust adaptive beamforming based on covariance matrix reconstruction with annular uncertainty set constraints

**DOI:** 10.1371/journal.pone.0327461

**Published:** 2025-07-21

**Authors:** Gaoxiang Xing, Zhixiang Yao, Hongkai Wei, Yunhua Hu

**Affiliations:** College of Electronics Engineering, Naval University of Engineering, Wuhan, Hubei, China; Whale Wave Technology Inc, CHINA

## Abstract

A robust adaptive beamforming method based on the interference covariance matrix reconstruction with annular uncertainty set constraints is proposed to address the issues of covariance matrix mismatch and steering vector mismatch caused by the distortion of flexible array shapes. The proposed method first eliminates the target components under the constraint of the annular uncertainty set to accurately re-construct the covariance matrix, and then corrects the steering vector using a constrained optimization method. This effectively reduces the impact of mismatch and significantly enhances the performance of the adaptive beamforming algorithm with array distortion, improving the robustness of the adaptive beamforming algorithm for flexible arrays. Simulation and sea trial data validate that the new method achieves a performance improvement of 4–10 dB.

## 1. Introduction

Adaptive beamforming [1–3] is capable of suppressing interference in complex environments and is widely be-ing applied in sonar systems. However, potential model mismatches during applications can lead to a degradation in performance, potentially to the level of conventional beamforming [4–9]. Taking towed linear arrays as an exam-ple, distortion of the array during being towed can result in mismatches in the covariance matrix and steering vec-tors, which in turn leads to a loss of performance in adaptive beamforming algorithms and a reduction in spatial processing gain.

In response to the covariance matrix mismatch, reference [10] proposes a method based on the Minimum Vari-ance Distortionless Response (MVDR) spatial spectrum search to reconstruct the covariance matrix. The method performs linear integration in the angular sector without the expected signals to reconstruct the covariance matrix. Although this significantly reduces the impact of the expected signal component, the performance of the beam-former is noticeably degraded when there is a bias in the direction of the incoming wave. On this basis, reference [11] introduces an improved matrix reconstruction method that corrects the steering vector in the direction of the interference, thereby reducing the impact of errors in the direction estimation of the incoming wave. To further en-hance the accuracy of covariance matrix reconstruction, reference [12] suggests first reconstructing the covariance matrix of the desired signal, and then constructing a blocking matrix to eliminate the desired signal from the co-variance matrix.

In addressing the issue of steering vector mismatch errors, reference [13] proposes a worst-case performance optimization algorithm that directly optimizes the weight vector with the objective function of minimizing the ar-ray output power. Reference [14] presents a covariance matrix fitting algorithm, treating the desired signal steering vector as a variable in the optimization process to find its optimal solution. To prevent the optimized steering vec-tor from deviating from the array manifold, Li et al. proposed a dual-constraint robust Capon beamforming algo-rithm [15], which improves the accuracy of the solution. Reference [16] orthogonally decomposes the steering vec-tor mismatch error and transforms it into a quadratically constrained quadratic programming problem through semi-definite programming, avoiding the difficulties of parameter selection.

The aforementioned studies have respectively conducted work on covariance matrix reconstruction and steer-ing vector optimization. In practical applications of towed linear arrays, both the covariance matrix and the steer-ing vector may experience model mismatches simultaneously. To address this situation, this paper applies annular uncertainty set constraints simultaneously to the reconstruction of the interference covariance matrix and the cor-rection of the steering vectors. This can effectively reduce the target components in the covariance matrix, enhance the accuracy of covariance matrix reconstruction, and effectively mitigate the impact of steering vector mismatch, thereby improving the robustness of adaptive beamforming.

## 2. Array signal model

**Fig 1:**
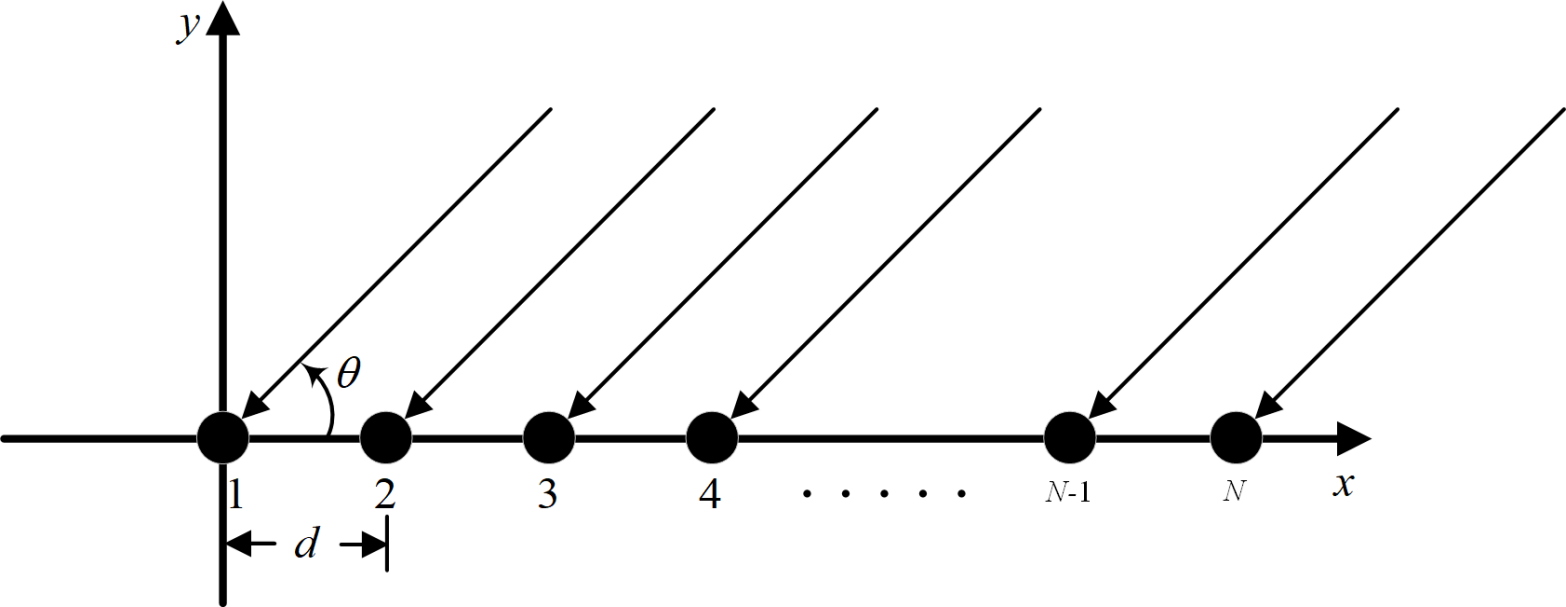
Schematic diagram of a uniform linear array.

Consider a uniform linear array (in short ULA) composed of *N* isotropic elements, with an inter-element spacing of *d*. Let the far-field signal be coplanar with the array (horizontal plane), and the positive *x*-axis direction is defined as 0° (see Fig 1). Without loss of generality, consider the semi-space of 0° to 180° for the ULA. The schematic diagram of a uniform linear array is shown in Fig 1.

Assuming the far-field signal contains 1 narrowband target and J narrowband interferences, with the narrowband target frequency being $f_{0}$ and the narrowband interference frequencies being $f_{j},j=1,2,\cdots ,J$. If the target and each interference are uncorrelated in each snapshot of the received signal, the array sampling data for the l-th snapshot is shown as follows:


$$\begin{array}{c} x(l)=a_{0}s_{0}(l)+\sum {\mathrm{\backslash nolimits}}_{j=1}^{J}a_{j}s_{j}(l)+n(l)l=1,2,\cdots ,L \end{array}$$ (1)

where, $a_{0}={\left[1,e^{-j2\pi f_{0}d{\cos\theta }_{0}/c},\cdots ,e^{-j2\pi f_{0}(N-1)d{\cos\theta }_{0}/c}\right]}^{T}$represents the steering vector of the target, $\theta_{0}$ is the direction-of-arrival of the target, c is the propagation speed of sound wave in water, $a_{j}={\left[1,e^{-j2\pi f_{j}d{\cos\theta }_{j}/c},\cdots ,e^{-j2\pi f_{j}(N-1)d{\cos\theta }_{j}/c}\right]}^{T}$ represents the steering vector of thej-th interference, j=1,2,⋯,J, $s_{0}(l)$ and $s_{j}(l)$ represent the target time series and the j-th interferer time series respectively, n(l) represents Gaussian white noise, x(l) represents the array sampling data [16].

The covariance matrix of array sampling data can be expressed as


$$R_{x}=E\left[x(l)x^{H}(l)\right]=R_{s}+R_{i}+R_{n}l=1,2,\cdots ,L$$ (2)

where E[·] denotes the mathematical expectation, $R_{s}$ represents the target covariance matrix, $R_{i}$ represents the interference covariance matrix, $R_{n}=\sigma_{n}^{2}I$ represents the noise covariance matrix.

## 3. Robust adaptive beamforming

Adaptive beamforming technology is the technology that an array to automatically adjust its parameters in response to changes in the environmental noise field, in order to adapt to the surrounding environment, suppress interference, and detect desired signals. In other words, the adaptive beamforming system can “learn” in real time, reducing the sensitivity of arrays to noise (including interference) while maximizing the sensitivity to signals. The optimal algorithm for adaptive beamforming is the MVDR algorithm.

### 3.1 Principle of MVDR

While finding direction by spatial spectrum estimation, the power of the signal source in a certain beam not only contributes to the direction of the incoming wave but also contributes to various degrees in other directions of the beam. In other words, the output power of the array includes not only the contribution of the signal from the designated direction but also contributions from other directions. Therefore, Capon proposed to minimize the overall output power while keeping the output power in the main lobe direction unchanged, which is called the minimum power estimator, also known as the MVDR beamformer [17]. This can be mathematically represented as


$$\min_{w}w^{H}R_{x}ws.t.\;w^{H}a_{0}=1$$ (3)

where $a_{0}$ is the steering vector on the target direction $\theta_{0}$, “s.t.” is the abbreviation “subject to”. Eq. (3) attempts to minimize the power contributed by noise and any interference from directions other than $\theta_{0}$, while ensuring that the signal power at the observation direction $\theta_{0}$ remains unchanged. Therefore, it can be considered as a sharp spatial bandpass filter. By applying the Lagrange multiplier method to solve the Eq. (3), the optimal beamforming weight vector is obtained by


$$w=\frac{R_{x}^{-1}a_{0}}{a_{0}^{H}R_{x}^{-1}a_{0}}$$ (4)

Considering the actual usage scenarios of towed linear arrays, it is not difficult to find that:

(1) The array received data covariance matrix calculating Eq. (2) indicates that a sufficient number of samples are needed to ensure the accuracy of the calculation. However, in sonar applications, due to the movement of both the towing ship and the targets, the snapshot number of available stable-state data is limited. Therefore, the Eq. (2) is usually estimated by a finite number of snapshots:

$$R_{x}=\frac{1}{L}xx^{H}$$ (5)

where L represents snapshot number.

(2) The covariance matrices calculated by Eq. (2) or Eq. (5) incorporates the target component, and its application to the MVDR algorithm leads to attenuation of the target’s signal intensity. Therefore, to enhance the robustness of the adaptive algorithms, it is desired that the covariance matrix does not contain the target components. This paper intends to adopt the interference covariance matrix reconstruction technology based on annular uncertainty set constraints to achieve this goal.
(3) In the operational context of a towed linear array, the array is subject to sway motion, which causes the steering vector of the array to deviate from its ideal value $a_{0}$. If the $a_{0}$ is used in Eq. (4) to get the weight vector, it will inevitably lead to errors in the weight vector, and consequently, the performance of the adaptive algorithm will decline. This paper intends to estimate the actual steering vector by a method based on uncertainty set constraints.

### 3.2 Interference covariance matrix reconstruction based on annular uncertainty set constraints

Assuming there is 1 target and J spatially separable interferences in the space, the target area is denoted as $\Theta_{S}$, the *j*-th interference area is denoted as $\Theta_{j}$ (j=1,2,⋯,J), and the total interference area is denoted as $\Theta_{J}=\Theta_{1}\cup \Theta_{2}\cup \cdots \Theta_{J}$. For the array’s observation space, such as the linear array observation space of 0° ~ 180° considered by this algorithm, the remaining area excluding the target area and interference area is considered to be approximately unaffected by the energy of the target and interference, which is referred to as the noise area $\Theta_{n}$ ($\Theta_{n}\text{= }{\overset{―}{\Theta_{J}\cup \Theta }}_{S}$).

Assuming that there is only noise and no targets present, the MVDR power in the direction θ is


$$p(\theta )=\frac{1}{a^{H}(\theta )R_{n}^{-1}a(\theta )}=\frac{1}{a^{H}(\theta )(\sigma_{n}^{2}I{)}^{-1}a(\theta )}=\frac{\sigma_{n}^{2}}{N}=\sigma_{r}^{2}$$ (6)

where $\sigma_{r}^{2}=\frac{\sigma_{n}^{2}}{N}$ represents the power of the residual noise [12]. Since the values of $\sigma_{r}^{2}$ and θ are unrelated, it is considered that the residual noise is uniformly distributed throughout the entire space.

**Fig 2:**
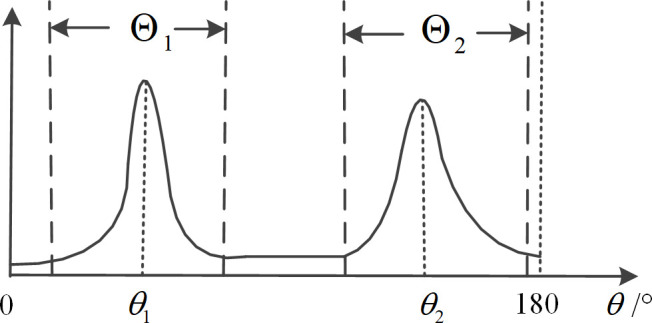
Schematic diagram of regional division under independent peak case.

Based on the preprocessing of peak energy detection via deconvolution [18,19], the division of the target area, interference area, and noise area is further improved. The criteria for dividing the areas are as follows; the corresponding diagrams are shown in Fig 2–Fig 4.

**Fig 3:**
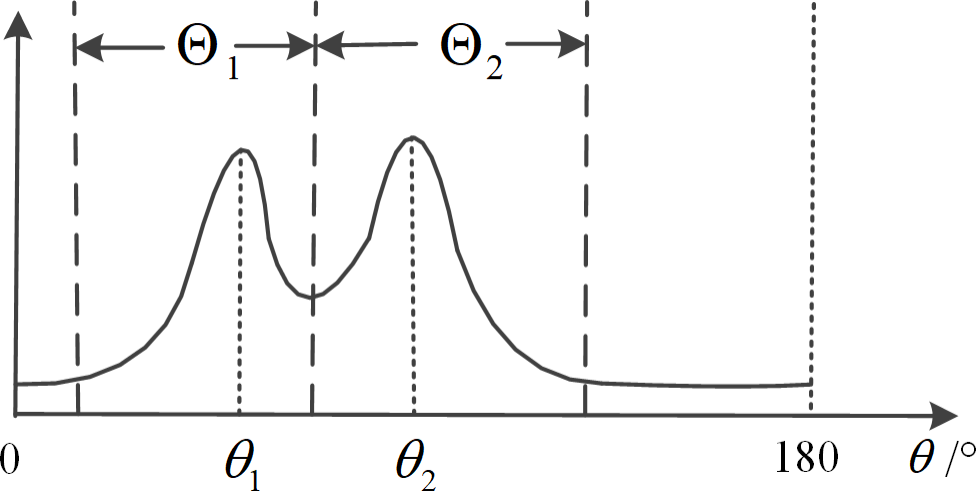
Schematic diagram of regional division under connected peaks case.

**Fig 4:**
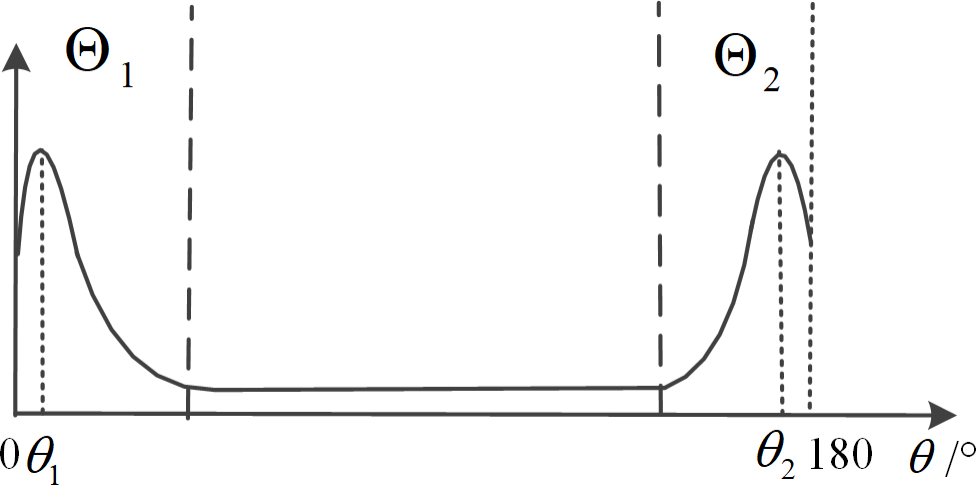
Schematic diagram of regional division under peak near the boundary case.

1. When the space power spectrum has nulls close to the power spectrum baseline, the target spectrum peaks are considered independent. The angular region between the two nulls is selected as the target angular region, which is shown in Fig 2.
2. When two or more targets are in close proximity in space, if there is a minimum value significantly above the power spectrum baseline between two peaks, this minimum value point is selected as the boundary between the two close targets, which is shown in Fig 3. At this time, if there is a valley close to the baseline value on the other side of the peak, the point where this valley located is used as the regional endpoint.
3. For spectrum peaks close to 0° or 180°, select 0° or 180° as one of the endpoints of that target’s region, which is shown in Fig 4.

Based on the preliminary estimated results of the direction of arrival (DOA)., each region is divided. In the already divided noise region $\Theta_{n}$, it can be approximately considered that there is no spatial signal source. Therefore, the average MVDR power on the noise region can be taken as the residual noise power $\sigma_{r}^{2}$, that is:


$$\sigma_{r}^{2}=\int_{\Theta_{n}}\frac{1}{a^{H}(\theta )R_{x}^{-1}a(\theta )}\;d\theta$$ (7)

It is assumed that within the divided regions, the interference leakage power $\sigma_{j}^{2}$ will not affect the target power estimation. Therefore, it is considered that the undesired disturbance power is approximately equal to the residual noise power $\sigma_{u}^{2}$:


$$\sigma_{u}^{2}=\sigma_{r}^{2}+\sigma_{c}^{2}\approx \sigma_{r}^{2}=\frac{\sigma_{n}^{2}}{N}$$ (8)

Approximating $\sigma_{u}^{2}=\sigma_{r}^{2}$, we can deduce the noise power to be $\sigma_{n}^{2}=N\sigma_{r}^{2}=N\sigma_{u}^{2}$. By integrating the MVDR power spectrum that eliminates undesired disturbances within the target region, the target covariance matrix can be reconstructed:


$${\hat{R}}_{s}=\int_{\Theta }\left(p(\theta )-\sigma_{u}^{2}\right)a(\theta )a^{H}(\theta )d\theta \;=\int_{\Theta }\left(\frac{1}{a^{H}(\theta )R_{x}^{-1}a(\theta )}-\sigma_{u}^{2}\right)a(\theta )a^{H}(\theta )d\theta$$ (9)

Perform an eigenvalue decomposition on the matrix ${\hat{R}}_{s}$ obtained from Eq. (9)


$$\begin{array}{c} {\hat{R}}_{s}=\sum {\mathrm{\backslash nolimits}}_{i=1}^{N}\gamma_{i}c_{i}c_{i}^{H} \end{array}$$ (10)

where $\gamma_{i}$ ($\gamma_{1}\geq \gamma_{2}\geq \cdots \geq \gamma_{N}$) represents the i -th eigenvalue of matrix ${\hat{R}}_{s}$, and $c_{i}$ represents the eigenvector corresponding to the eigenvalue $\gamma_{i}$ (i=1,2,⋯N).

Let ${𝐁}_{h}$ represent the set of eigenvectors corresponding to the principal eigenvalues. Construct a subspace by ${𝐁}_{h}$, and then use the projection complement matrix of this subspace as the blocking matrix:


$$P_{B}=P_{B}^{H}=I-{𝐁}_{h}B_{h}^{H}$$ (11)

The projected value of the sampled covariance matrix $R_{x}$ onto the subspace spanned by $P_{B}$ is $P_{B}^{H}R_{x}P_{B}$, which can be considered to contain no target components. Therefore, the above process relies on the blocking matrix $P_{B}$ to eliminate the target components from $R_{x}$. After further eliminating the noise projection components, the projected interference covariance matrix ${\hat{R}}_{i}$ is obtained as follows:


$${\hat{R}}_{i}=P_{B}^{H}R_{x}P_{B}-\sigma_{n}^{2}P_{B}^{H}P_{B}$$ (12)

Given a significant difference between ${\hat{R}}_{i}$ and $R_{x}$, and that $P_{B}$ is the projection complement matrix of the subspace ${𝐁}_{h}$, $P_{B}^{H}R_{x}P_{B}$ will not alter the magnitude of the interference eigenvalues within $R_{x}$. Therefore, only the eigenvalues of ${\hat{R}}_{i}$ are taken as the interference power for constructing the covariance matrix. Perform an eigenvalue decomposition on ${\hat{R}}_{i}$:


$$\begin{array}{c} {\hat{R}}_{i}=\sum {\mathrm{\backslash nolimits}}_{j=1}^{J}\gamma_{j}c_{j}c_{j}^{H}={\hat{A}}_{i}\hat{\Lambda }{\hat{A}}_{i}^{H} \end{array}$$ (13)

where $\gamma_{j}$ represents the eigenvalue of ${\hat{R}}_{i}$, $c_{j}$ represents the eigenvector corresponding to $\gamma_{j}$, ${\hat{A}}_{i}$ denotes the set of interference steering vectors, ${\hat{A}}_{i}=[{\hat{a}}_{1},{\hat{a}}_{2},\cdots {\hat{a}}_{J}]$, where ${\hat{a}}_{j}$ represents the estimated steering vector of the *j*-th interference. $\hat{\Lambda }$ represents a diagonal matrix with the main diagonal elements being the interference power $\sigma_{j}^{2}$. Given the a priori about the approximate direction of the interference, one can determine ${\hat{a}}_{j}$ and ${\hat{A}}_{i}$, and then, in conjunction with ${\hat{R}}_{i}$, get the interference power diagonal matrix $\hat{\Lambda }$ as follows:


$$\hat{\Lambda }=({\hat{A}}_{i}^{H}{\hat{A}}_{i}{)}^{-1}{\hat{A}}_{i}^{H}{\hat{R}}_{i}{\hat{A}}_{i}({\hat{A}}_{i}^{H}{\hat{A}}_{i}{)}^{-1}\;=({\hat{A}}_{i}^{H}{\hat{A}}_{i}{)}^{-1}{\hat{A}}_{i}^{H}(P_{B}^{H}R_{x}P_{B}-\sigma_{n}^{2}P_{B}^{H}P_{B}){\hat{A}}_{i}({\hat{A}}_{i}^{H}{\hat{A}}_{i}{)}^{-1}$$ (14)

The Eq. (14) provides an estimation for the interference power, and it is clear that the estimate of $\hat{\Lambda }$ will be directly affected by ${\hat{A}}_{i}$. At this point, the ${\hat{A}}_{i}$ is gotten based on the ideal array parameters, and its mismatch error will increase as the array model mismatch becomes more and more severe.

The true interference steering vector $a_{j}$ and the estimated interference steering vector ${\hat{a}}_{j}$ have a mismatch error, which is represented by the error vector e. Therefore, the true value of the interference steering vector is $a_{j}={\hat{a}}_{j}+e$. A spherical uncertainty set model [20–22] is established, which assumes that the true value of $a_{j}$ exists within the model range centered at the estimated ${\hat{a}}_{j}$, where ε represents the upper bound of the steering vector error e, also known as the radius of the spherical uncertainty set, such that


$${\left\|a_{j}-{\hat{a}}_{j}\right\|}^{2}\leq \varepsilon$$ (15)

**Fig 5:**
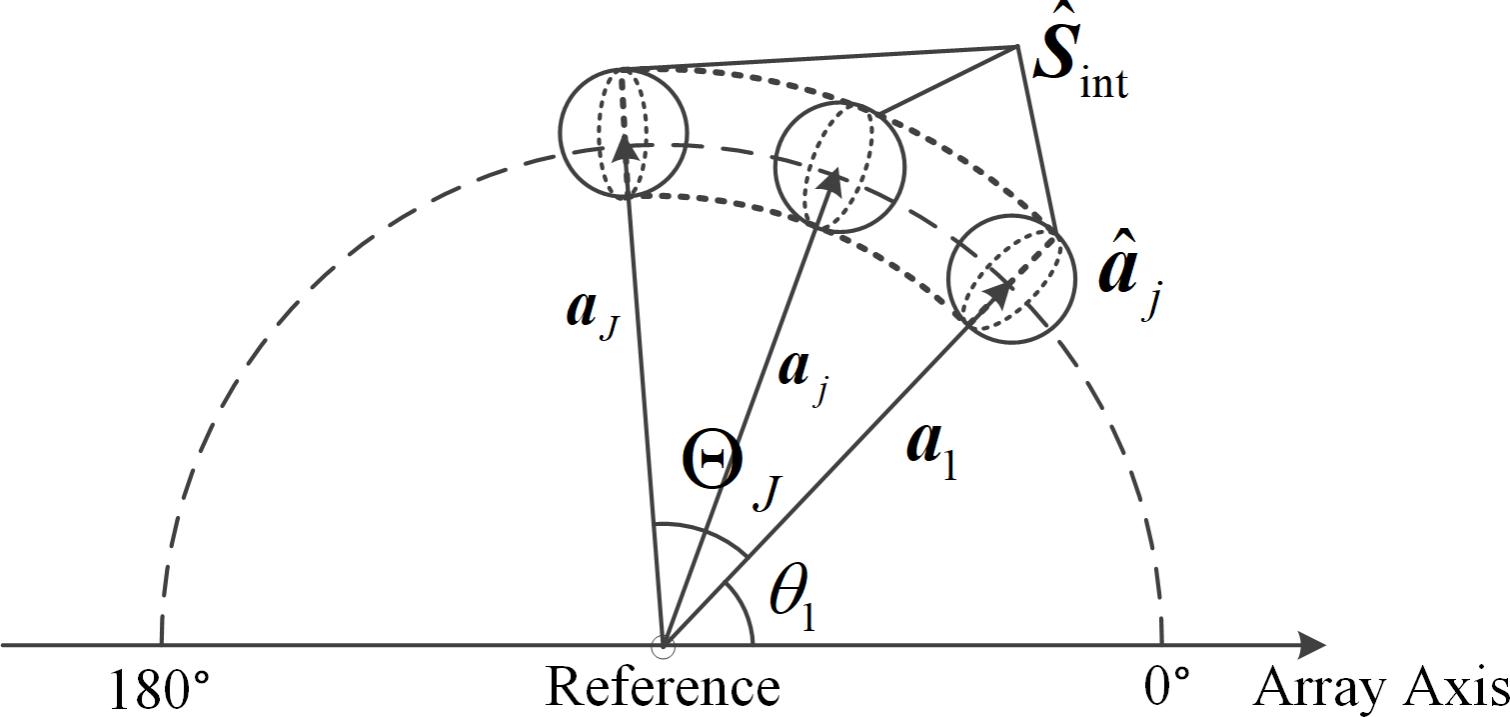
Schematic diagram of an annular uncertainty set.


It can be considered that each interference steering vector within the interference region $\Theta_{J}$ is constrained within a spherical uncertainty set centered on itself. Across the entire interference region, these spherical uncertainty sets collectively form an annular set, as shown in Fig 5. Therefore, it is believed that $a_{j}$ exists within the following annular uncertainty set:

$${\hat{S}\left\{a_{j}|a_{j}={\hat{a}}_{j}+e\;,\;\left\|e\right\|\;2\leq \varepsilon \text{ , }j=1,2,\cdots J\right\}}_{int}$$ (16)

The optimization of the interference steering vector can be formulated as the following problem:


$$\underset{a_{j}}{min\;}a_{j}^{H}R_{x}^{-1}a_{j}\text{ , s.t. }{\left\|a_{j}-{\hat{a}}_{j}\right\|}^{2}\leq \varepsilon ,\;a_{j}\in {\hat{S}}_{int}$$ (17)

The Eq. (17) can be solved using optimization tools to obtain the corrected set of interference steering vector estimates, denoted as $A_{i}=\left[a_{1},a_{2},\cdots a_{J}\right]$.

The estimated interference steering vector $a_{j}$ obtained from the solution is used to reconstruct the set of corrected interference steering vectors $A_{i}$. The interference power diagonal matrix Λ is then deduced from $A_{i}$:


$$\Lambda =(A_{i}^{H}A_{i}{)}^{-1}A_{i}^{H}(P_{B}^{H}R_{x}P_{B}-\sigma_{n}^{2}P_{B}^{H}P_{B})A_{i}(A_{i}^{H}A_{i}{)}^{-1}$$ (18)

The interference power diagonal matrix Λ is solved based on the corrected steering vector $A_{i}$, which reduces the impact of steering vector mismatch error. Its main diagonal elements can more accurately represent the interference power values [23]. By taking the main diagonal elements of Λ as the interference power, the interference covariance matrix is reconstructed as follows:


$${\hat{\hat{R}}}_{i}=A_{i}diag(\Lambda )A_{i}^{H}$$ (19)

where “ diag(·) ” denotes the operation of extracting the diagonal elements of a matrix.

Combining the $\sigma_{n}^{2}$ obtained already, the interference-plus-noise covariance matrix is reconstructed as follows:


$${\hat{R}}_{\text{i+n}}={\hat{\hat{R}}}_{i}+{\hat{R}}_{n}=A_{i}diag(\Lambda )A_{i}^{H}+\sigma_{n}^{2}I$$ (20)

### 3.3 Robust adaptive beamforming based on uncertainty set constraints

Based on the concept of Covariance Matrix Fitting (CMF) [14], construct a target component matrix to fit the sampled covariance matrix $R_{x}$, assuming the target’s power to be $\sigma^{2}$, with $\max{\;\sigma }^{2}\text{ , s.t. }R_{x}-\sigma^{2}{\hat{a}}_{s}{\hat{a}}_{s}^{H}\geq 0$. Considering that the true target power $\sigma_{s}^{\text{2}}$ cannot be obtained to replace $\sigma^{2}$, the constrained problem should be transformed. Reference [14] points out that treating $\sigma^{2}{\hat{a}}_{s}{\hat{a}}_{s}^{H}$ as a matrix $R_{0}$ of the same dimension as $R_{x}$.The problem finding the maximum value of $\sigma^{2}$ can be transformed into that finding the minimum value of $\sigma^{2}{\hat{a}}_{s}{\hat{a}}_{s}^{H}$. The above model can be rewritten as follows:


$$min\;{\hat{a}}_{s}^{H}R_{0}^{-1}{\hat{a}}_{s}\text{ , s.t. }R_{x}\geq R_{0}$$ (21)

Assuming the mismatch error between the true steering vector $a_{s}$ and the estimated steering vector ${\hat{a}}_{s}$ is $e^{\prime }$, $a_{s}$ exists within a spherical uncertainty set $S_{a}$ centered on ${\hat{a}}_{s}$. The constrained set is represented as:


$$S_{a}=\left\{a_{s}|a_{s}={\hat{a}}_{s}+e^{\prime }\;,\;\left\|e^{\prime }\right\|\;2\leq \varepsilon^{\prime }\right\}$$ (22)

The Eq. (21) can be rewritten as


$$\min_{{\hat{a}}_{s}}{\hat{a}}_{s}^{H}R_{0}^{-1}{\hat{a}}_{s}\text{ , s.t.}\left\{ \begin{array}{c} R_{x}\geq R_{0}\;\\ \;{\left\|a_{s}-{\hat{a}}_{s}\right\|}^{2}\leq \varepsilon^{\prime } \end{array}\right.\;$$ (23)

Substituting the corrected ${\hat{a}}_{s}$ and ${\hat{R}}_{\text{i+n}}$ obtained from the annular uncertainty set constraints into the MVDR weight vector solution formula yields the weight vector of the Annular Uncertainty Set Constraint Covariance Matrix Reconstruction (AuscCMR) algorithm.


$$w_{AuscCMR}=\frac{{\hat{R}}_{\text{i+n}}^{-1}{\hat{a}}_{s}}{{\hat{a}}_{s}^{H}{\hat{R}}_{\text{i+n}}^{-1}{\hat{a}}_{s}}$$ (24)

### 3.4 Algorithm implementation steps

The specific implementation steps of the AuscCMR algorithm are summarized as follows:

1. Filter the sampled array receiving data $x_{B}$, and divide it into M subbands $\left[x_{b1},x_{b2},\cdots x_{bM}\right]$.
2. Perform conventional beamforming (CBF) on the subband signals (taking the $x_{bm}$ as an example), obtain its spatial spectrum, denoted as $p_{bm}$.
3. Optimize the spatial spectrum $p_{bm}$ by a deconvolution peak energy detection algorithm to obtain the optimized spatial spectrum, denoted as $p_{bm-dCv}$.
4. Perform spectral peak search on $p_{bm-dCv}$, and use the results as directions of spatial signal sources so as to divide the angular ranges, such as Θ, $\Theta_{J}$, and $\Theta_{n}$.
5. Based on the AuscCMR, calculate ${\hat{R}}_{\text{i+n}}$ and ${\hat{a}}_{s}$, and then use Eq. (24) to solve for $w_{AuscCMR}$.
6. Get the subband spatial spectrum based on $w_{AuscCMR}$ and $x_{bm}$.
7. Add the spatial spectrums of the M subbands to obtain the spatial spectrum of $x_{B}$.

Superimpose the spatial spectrum obtained from multiple frames to obtain a bearing-time recorder diagram of the received signal over time and estimate the direction of the target.

## 4. Performance analysis

### 4.1 Simulation data analysis

Consider a 20-element uniform linear array with design frequency 750 Hz, arranged by half-wavelength spacing. Assume a stationary target is located at 50°, and an interferer with an initial bearing of 100° moves towards the endfire of the array with a rate of bearing variation 0.1 °/s. The signal-to-noise ratio (SNR) is 0 dB, the interference-to-noise ratio (INR) is 20 dB, and both the target and interference have a frequency of 750 Hz. The sampling snapshot number is 200, and Gaussian white noise is superimposed across the entire frequency band. The frequency band of the array is set to 650 Hz-850 Hz, with each subband width 40 Hz. Then the whole band is divided into 5 subbands, and the FIR filter order is set to 128. The total duration of the array received data is 200 seconds, with each frame lasting 1 second.

For a towed linear array working in low-frequency, under the working condition where the hull of the ship serves as the towing platform, the phase deviation caused by the vertical distortion of the towed array can be neglected. The distortion in the horizontal direction will cause a change in the position of the array elements [24], leading the array to change from a uniform linear array to a non-uniform curved array. Reference [24] provides a recursive formula for the coordinates of the array elements in the case of a cosine-type distortion:


$$\begin{array}{c} \;y_{n}=\alpha \cos\rho x_{n}\\ \{\begin{array}{c} {}*20l\Delta x_{n+1}=\frac{d}{\sqrt{1+\alpha^{2}\rho^{2}\sin^{2}\rho x_{n}}}\\ x_{n+1}=x_{n}+\Delta x_{n+1},x_{0}=0 \end{array}(n=1,2,\cdots N) \end{array}$$ (25)

where the cosine amplitude α and the cosine period ρ are used to characterize the degree of array curvature.

**Fig 6:**
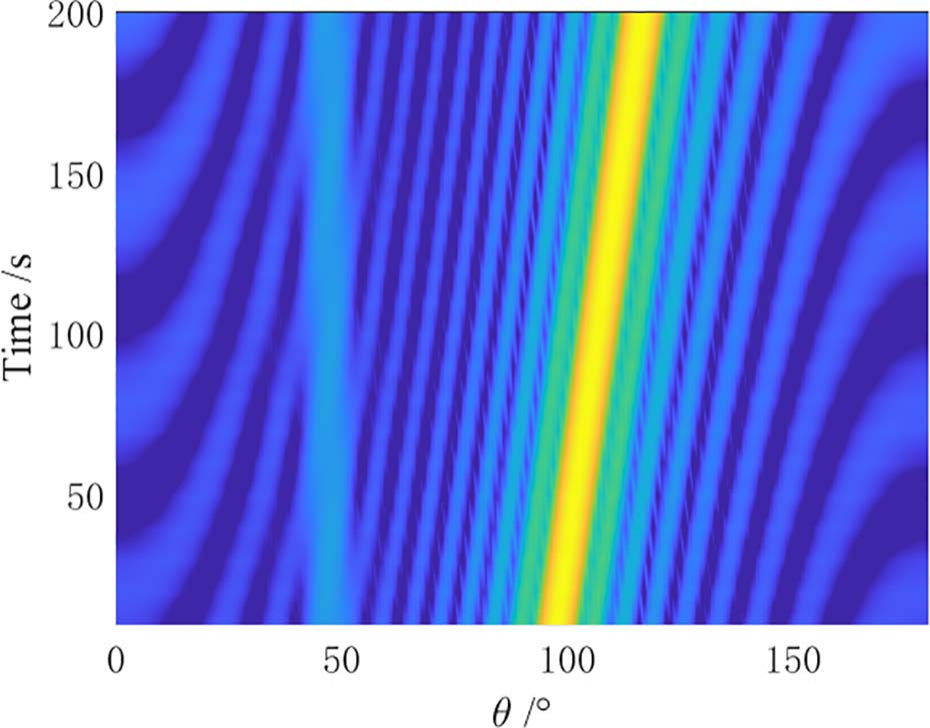
BTR Chart of CBF Under Array Distortion Conditions (α=2λ, ρ=4N).

In the simulation, a towed linear array with a swing amplitude α=2λ and a swing period ρ=4N is set for the array distortion. Comparative simulation experiments are conducted by the CBF algorithm, MVDR algorithm, residual noise elimination (RNE) algorithm [12], CMF algorithm [14], and AuscCMR algorithm. The robustness of each algorithm is analyzed, and the bearing-time recorder (BTR) charts within 200 seconds are shown in Fig 6–Fig 10.

**Fig 7:**
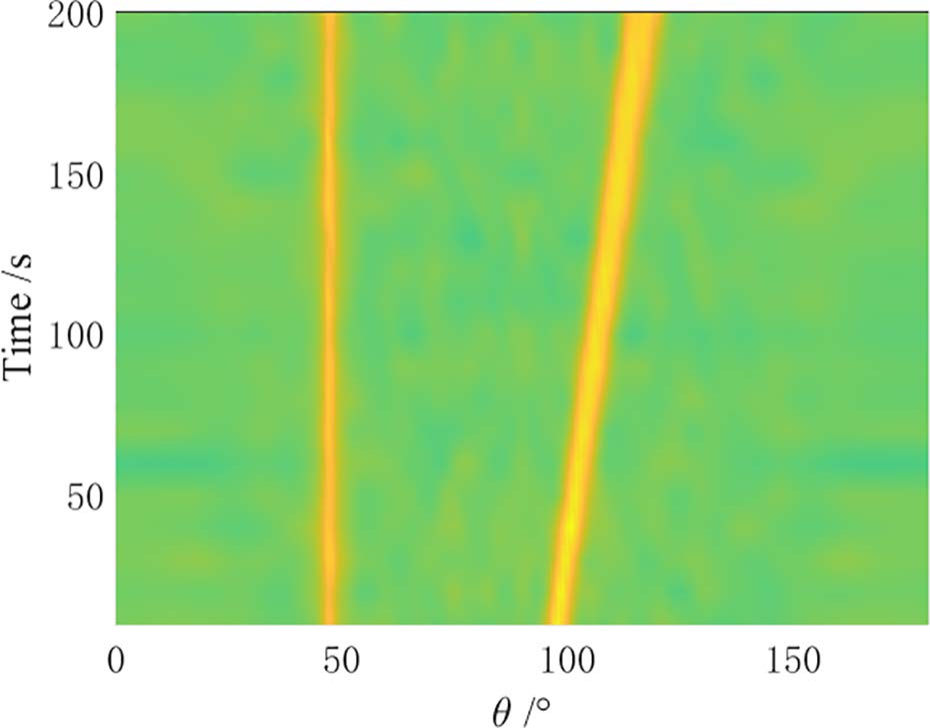
BTR Chart of MVDR Under Array Distortion Conditions (α=2λ, ρ=4N).

**Fig 8:**
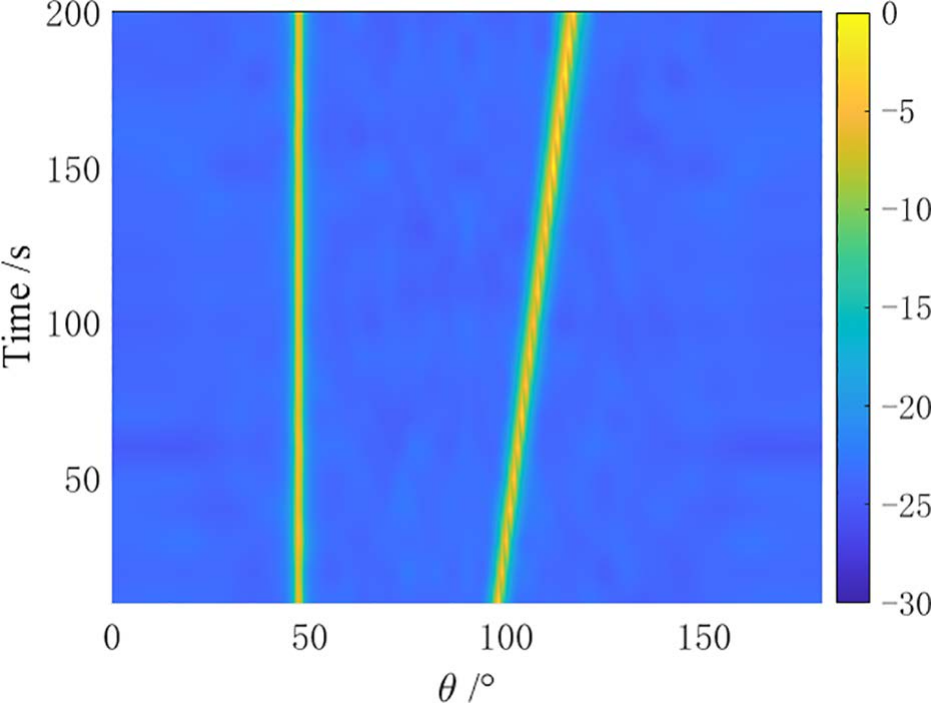
BTR Chart of CMF Under Array Distortion Conditions (α=2λ, ρ=4N).

**Fig 9:**
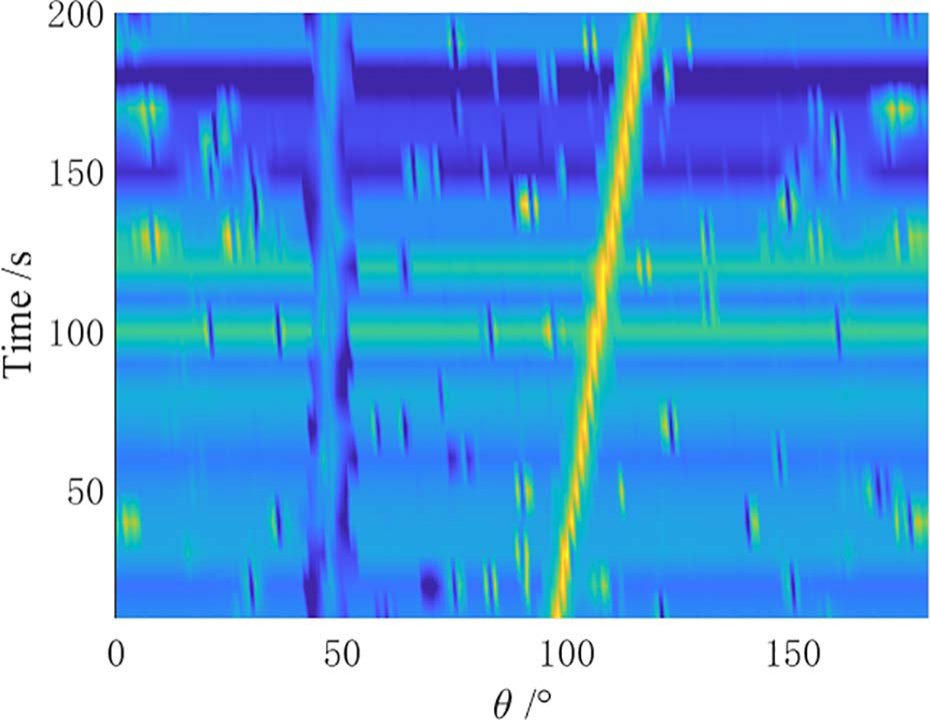
BTR Chart of RNE Under Array Distortion Conditions (α=2λ, ρ=4N).

**Fig 10:**
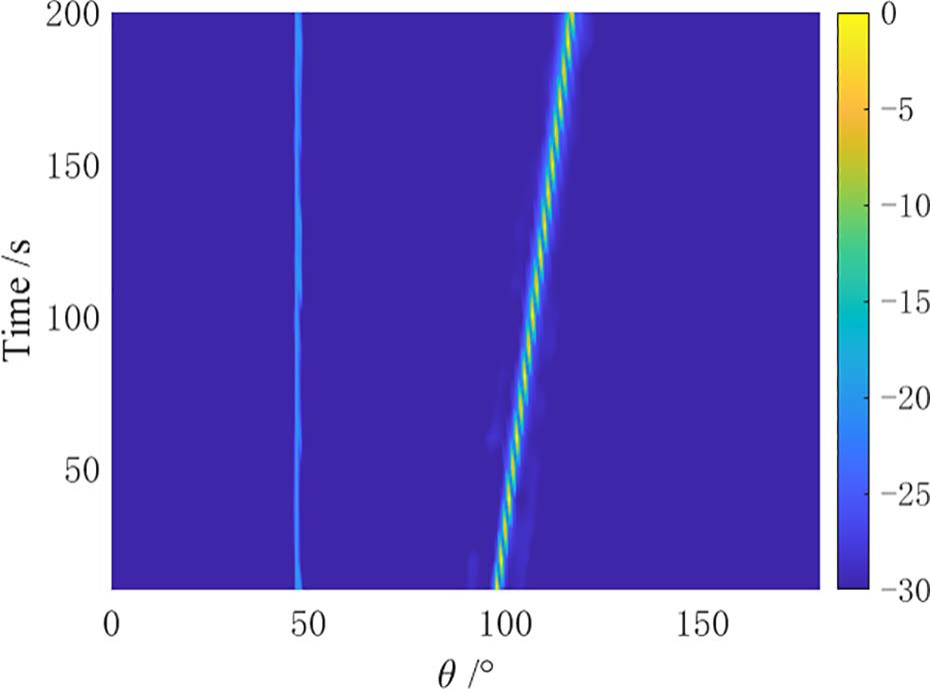
BTR Chart of AuscCMR Under Array Distortion Conditions (α=2λ, ρ=4N).

As shown in Fig 6 – Fig 10, under simulation conditions of array formation bending, the AuscCMR algorithm demonstrates significant advantages. It provides a clear trajectory of the target with minimal background fluctuations. This algorithm significantly enhances the beamformer’s anti-interference capabilities through steering vector constraints and the elimination of undesired disturbances. It exhibits a strong suppression ability against interference and noise, with a narrow beam and distinct peak direction, making it easy to estimate the directions of targets.

**Fig 11:**
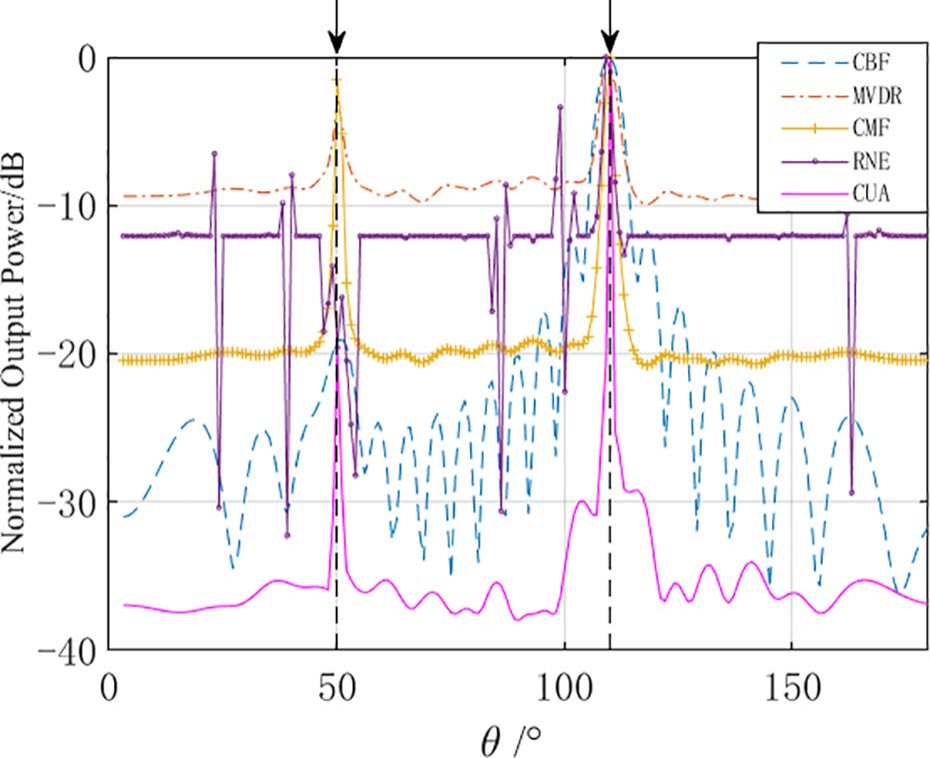
Spatial spectrum of the 100th frame under array distortion conditions (20 elements).

Analyze the 100th frame of the bearing-time recorder chart. The spatial spectrum at that moment is shown in Fig 11.

Analyzing Fig 11, it can be seen that under the condition of array distortion error, the AuscCMR algorithm can accurately output the signal power, demonstrating more than 20 dB difference in signal power. The noise floor and sidelobe levels are the lowest, and the beampattern is narrowest. The AuscCMR algorithm possesses significant robustness with its high accuracy in reconstructing matrices and corrected steering vectors.

### 4.2 Sea trial data verification

**Fig 12:**
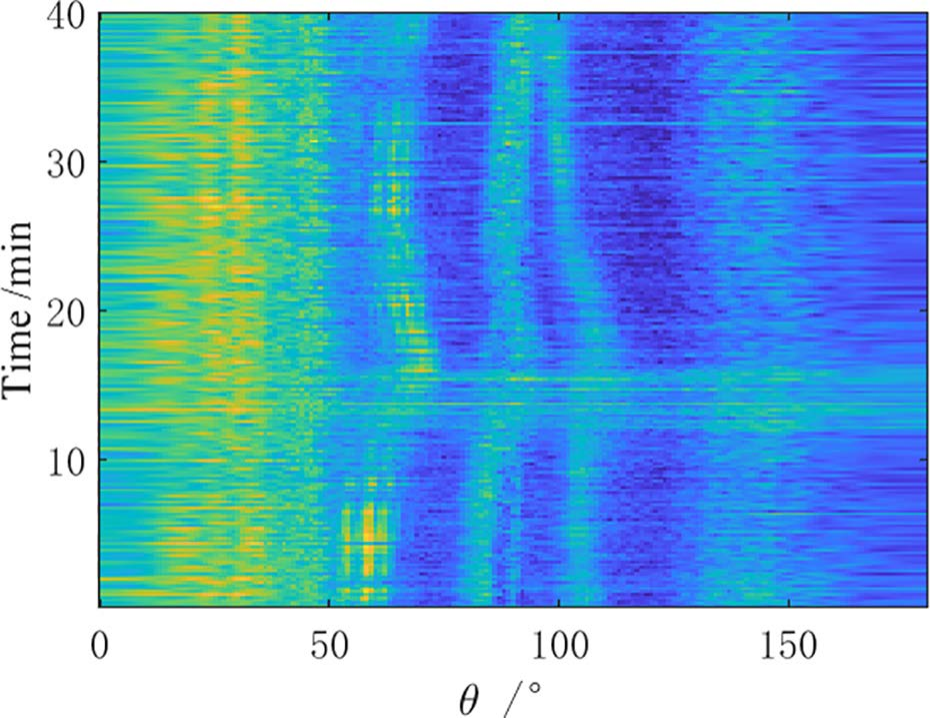
BTR of sea trial data by CBF.

A measurement experiment was conducted in a certain sea area in the South China Sea using a certain type of towed linear array sonar in April, 2021. During the observation time, the towing ship performed a small angle turning maneuver while sailing, and then continued straight-line navigation. Direction finding tests were conducted on two preset distant-fixed acoustic targets (①, ②). The bearing-time recorder chart obtained from the analysis of the received data by the towed array sonar within 40 minutes is shown in Fig 12–Fig 14.

**Fig 13:**
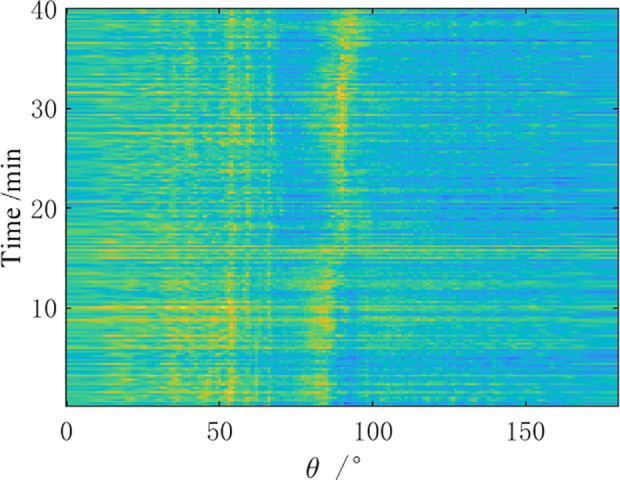
BTR of sea trial data by MVDR.

**Fig 14:**
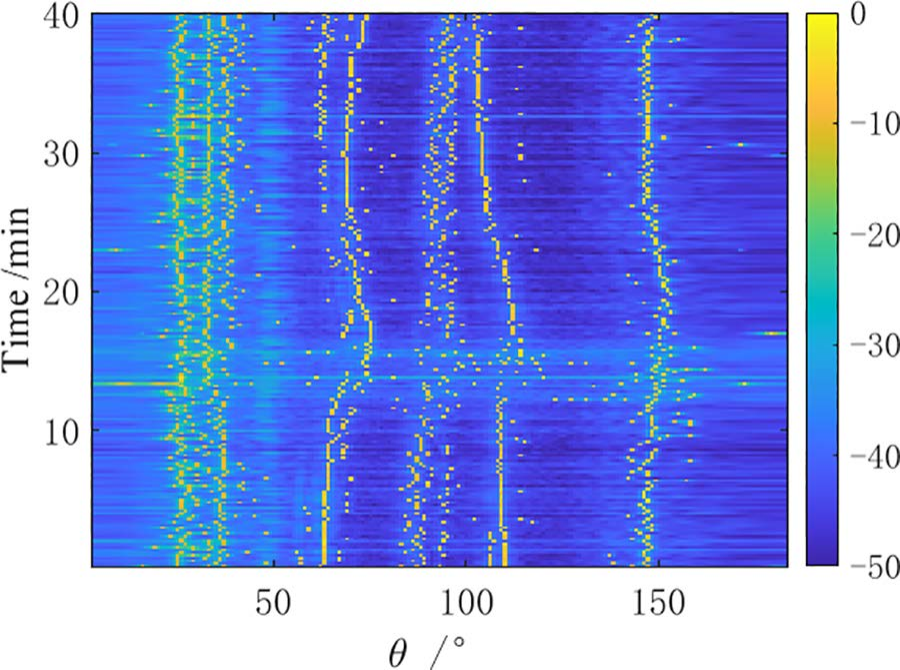
BTR of sea trial data by AuscCMR.

As shown in Fig 12–Fig 14, the bearing-time recorders indicate that the AuscCMR algorithm has a clear trajectory and significant interference suppression effects. Based on the CBF spatial spectrum, the signal direction is preliminarily estimated using the deconvolution peak energy detection algorithm, providing more accurate a priori information for the beamformer. This makes the interference power estimation and covariance matrix reconstruction of the AuscCMR algorithm more accurate, greatly enhancing its interference suppression capability. As a result, the background of AuscCMR in the BTRs is the lowest and the fluctuations are the smallest. At the same time, the correction of the steering vector’s uncertainty set constraint makes the AuscCMR algorithm more clearly distinguish strong signal sources within the range of 0° to 50° and near the direction 150°, reducing the sidelobe effects and lowering the difficulties of bearing estimation for weak signals.

**Fig 15:**
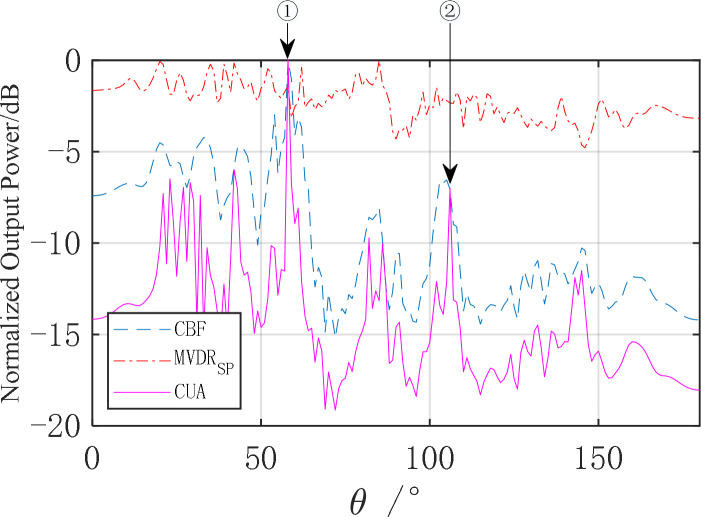
Spatial spectrum of the 300th second of sea trial data.

Analyze the spatial spectrum at a specific moment (taking the 300th second as an example), and its normalized spatial spectrum is shown in Fig 15.

As shown in Fig 15, under relatively complex ocean environmental conditions, the AuscCMR algorithm has more distinct peaks in the directions of ① and ②, with a significant reduction in the noise floor, achieving about a 10 dB improvement in signal-to-noise ratio (SNR) compared to the MVDR algorithm. Compared to the CBF algorithm, the AuscCMR algorithm has a narrower beamwidth, higher angular resolution, and clearer peak direction, with an approximate 4 dB improvement in SNR. The results of sea trial data processing indicate that the AuscCMR algorithm has strong robustness under conditions of towed array distortion.

## 5. Conclusions

This paper first uses a deconvolution peak energy detection method to achieve an angular preliminary estima-tion of the spatial spectrum energy peaks, which is used to divide the angular range. Then, it employs a designed annular uncertainty set constraint interference covariance matrix reconstruction algorithm to estimate the inter-ference-plus-noise covariance matrix and the steering vector. Based on these, it calculates a pseudo-MVDR weight vector to obtain the AuscCMR algorithm. The new algorithm corrects the interference estimation bias caused by array shape mismatch in covariance matrix reconstruction algorithms. At the same time, it uses a blocking matrix to eliminate the target components in the reconstructed matrix in advance, making the interference power estima-tion more accurate and significantly enhancing the robustness of the adaptive algorithm. Simulation and sea trial data verification show that the AuscCMR algorithm has a performance improvement of 4–10 dB.
