# Supplementary material for: Robust adaptive beamforming based on covariance matrix reconstruction with annular uncertainty set constraints
Source: PLoS One. 2025 Jul 21;20(7):e0327461. doi: 10.1371/journal.pone.0327461 (PMC12279156; doi:10.1371/journal.pone.0327461)
Supplement: Attachment — Submitted filename: Response to Reviewer2.docx [file pone.0327461.s002.docx]

Dear Reviewer #2

We sincerely thank you for your meticulous review of our paper. Your valuable suggestions have played a crucial role in enhancing the quality of this academic work.

|  | Reviewer #2’s Feedback | Answers |
| --- | --- | --- |
| 1 | The phrase "with-the-array-axis·as·0" is unclear. Based on Figure 1, this phrase should be "The x-axis positive direction is defined as 0° (see Figure 1)." | Thank you for your thorough review.  Replaced by “and the positive *x*-axis direction is defined as 0° (see Figure 1)” |
| 2 | Some mathematical symbols lack clear definitions. The symbol "E" in Equation (2) is undefined. "\(\sigma_u^2\)" in Equation (8) requires explicit definition. The term "diag" in Equation (19) needs explanation. | Thank you for your thorough review.  The authors have revised the manuscript in accordance with the reviewer’s comments and conducted a comprehensive linguistic refinement of the entire text to ensure terminological consistency and clarity of expression. |
| 3 | The English expression in the paper needs further improvement | Thank you for your thorough review.  A comprehensive linguistic refinement has been performed on the entire manuscript. |

Author	Gaoxiang Xing
